# Participatory development of evidence-based patient narrative videos for patients with eating disorders: a methodological approach and pilot data

**DOI:** 10.1186/s40337-024-01146-1

**Published:** 2024-11-20

**Authors:** Melissa-Claire Daugelat, Bettina Gregg, Sophia Helen Adam, Kathrin Schag, Joachim Kimmerle, Katrin Elisabeth Giel

**Affiliations:** 1 Department of Psychosomatic Medicine and Psychotherapy, Medical University Hospital Tübingen, Osiander str. 5, 72076 Tübingen, Germany; 2 Centre of Excellence for Eating Disorders Tübingen (KOMET), Tübingen, Germany; 3 Lived Experience Representative, Tübingen, Germany; 4 Department of Psychology, Eberhard-Karls-University Tübingen, Tübingen, Germany; 5 Leibniz-Institut Für Wissensmedien, Tübingen, Germany; 6 DZPG (German Centre for Mental Health), Tübingen, Germany

**Keywords:** Eating disorders, Motivation, Help-seeking, Lived experience, Patient involvement, Patient narratives, Recovery stories, Treatment

## Abstract

**Background:**

Patient narratives can best be defined as personal stories of persons previously or currently affected by a physical or mental health disorder. The collaborative development and implementation of such narratives reflects a participatory approach between researchers, patients, and members of the public towards the development of new interventions. Patient narratives can foster feelings of support and belonging, as well as increase hope and motivation towards recovery. Aims of this pilot study were (a) the collaborative development of a series of evidence-based patient narrative videos about eating disorders, (b) their initial evaluation with a group of participants without (current) eating disorders, and (c) to provide a reproducible documentation of this methodological approach.

**Method:**

A multi-stage participatory process was used including a) a systematic review, b) focus groups with affected persons, c) the participatory narrative development, and d) an initial pilot study with participants without (current) eating disorders. A former and currently recovered patient was recruited as a lived experience representative, while a psychotherapist provided the same information from a professional perspective. Control group videos featured the lived experience representative discussing a somatic condition unrelated to eating disorders (i.e., torn knee ligament). Two videos were created for each perspective with varying degrees of emotionality of the content.

**Results:**

Nineteen female participants without (current) eating disorders were recruited for the pilot study. All videos received positive ratings, however, participants rated videos in which the lived experience representative discussed her eating disorder as significantly more authentic than the control group videos, as well as significantly more empathic, useful, and better overall than the psychotherapist and control group videos. Participants further indicated a clear preference for videos with higher emotionality, regardless of which perspective or disorder was being presented.

**Discussion:**

The use of patient narratives for eating disorders is a relatively new methodological approach. This paper provides one example of how evidence-based patient narratives can be constructed. The patient narratives created in this study received positive feedback from participants without (current) eating disorders and are currently being tested in a 4-arm randomised controlled pilot study with patients affected by eating disorders.

## Introduction

The treatment of eating disorders (ED) is influenced by a myriad of personal, social, and environmental factors. In particular, a systematic review completed by our research group showed that patients most frequently report stigma, shame, and guilt as barriers to treatment, while caregivers and healthcare professionals report a lack of training for clinicians regarding ED to be a hindrance to effective treatment [1]. Meanwhile, one of the most prominent factors influencing the initiation of ED treatment is social support, or rather the lack of positive social support commonly found among persons affected by ED [1, 2]. Patients with ED typically report relying on the social support of their immediate family, however, some patients may also seek social support through friends, co-workers, or by engaging with other affected persons and their stories (e.g. [3]).

Patient narratives can be defined as personal stories presented by persons previously or currently affected by a physical or mental health disorder, and frequently comprise insights into the narrator’s challenges, struggles, and ultimately successes as they navigate treatment for their disorder [4, 5]. Patient narratives may be live, such as in peer-to-peer relationships, or else may be documented in a variety of mediums, including written texts such as books or blogs, documentary films, or videos posted on various social media platforms [5–7], and are therefore easily accessible to affected persons. Patient narratives can foster empathy and feelings of support and belonging [5, 8, 9], while showing that recovery is possible and providing currently affected persons with feelings of hope regarding their own recovery [10]. To date, only a handful of studies have examined the effects of patient narratives for patients with ED. A recent study by Gumz et al. [11] assessing factors influencing treatment uptake found that patients with anorexia nervosa who had reported reading and/or viewing patient narratives also reported significantly shorter durations of untreated ED (DUED), i.e. the amount of time that elapses between the onset of ED symptoms and treatment engagement [12]. Another study which retrospectively asked female patients with ED about their experiences with written patient narratives found that while patients in recovery benefited from engagement with patient narratives, patients who were not receiving treatment at the time of engagement were increasingly at risk for developing harmful social comparisons or “copy-cat” behaviours on the basis of the narratives [13]. Dawson et al. [10] further used patient interviews to create a series of written patient narratives, in which such possibly triggering or adverse contents were eliminated. Women with anorexia nervosa were recruited for the study and sent the patient narratives to read over a two-week period. While the results of this study showed no short-term effects on current patients’ motivation or self-efficacy, qualitative results showed that the patient narratives were well accepted and that participants would recommend their use for other patients [10].

Studies examining the use of patient narrative videos for other mental disorders, including depression and anxiety, found that engaging with at least one patient narrative video resulted in significant long-term increases in participants’ reported quality of life and meaning of life [14]. Similar results have also been found in the field of psychosis, where viewing patient narrative videos resulted in increased self-efficacy and hope towards one’s own recovery [15, 16]. Interestingly, when presented with nearly identical content from a healthcare professional’s point of view, these effects remained but were significantly reduced [15], suggesting that while content itself may evoke positive effects, the peer element of patient narratives appears to be an important component of these videos.

The process of developing patient narratives videos is a relatively new methodological approach and little information has been documented regarding how patient narratives should be developed in order to be most beneficial for the target population of affected persons. Previous work completed by LaMarre and Rice [17] focused on a digital storytelling workshop, in which participants co-created a series of videos focusing on ED recovery. These workshops consisted of five key components, including an introduction to the context, a story circle to develop and share ideas, technical introductions, an open studio for content production, and a screening and reflection of the videos. The videos created in the workshop were later used by participants for a variety of personal and/or public purposes (e.g., sharing on social media platforms) [17]. This paper aims to further develop the methodology of patient narratives and to document the process of developing evidence-based patient narrative videos for persons affected by ED.

## Study aims

Patient and Public Involvement (PPI) refers to the collaboration between researchers, patients, and members of the public when planning, conducting, or disseminating clinical research studies [18]. Through the use of PPI, affected individuals actively contribute to the development of new interventions that are relevant to the target patient group [19, 20]. The current study therefore incorporated PPI throughout the development and assessment of a series of evidence-based patient narrative videos for patients with ED. The videos featured three varying perspectives from a) a lived experience representative discussing her experiences with an ED (Video 1A and Video 1B), b) a psychotherapist (Video 2A and Video 2B), and c) the lived experience representative reporting on a somatic condition unrelated to ED (Video 3A and Video 3B). The videos further varied based on the level of emotionality of the content presented, with one half of the videos (i.e., video versions A) featuring content with a low degree of emotionality, and the other half of the videos (i.e., video versions B) featuring content with a higher degree of emotionality. A pre-pilot study with a student population was completed to gain initial insights into the possible acceptability and feasibility of these patient narrative videos, as well as to determine which videos would be most suitable for a larger pilot study with affected persons with ED.

## Method/design

**Fig. 1:**
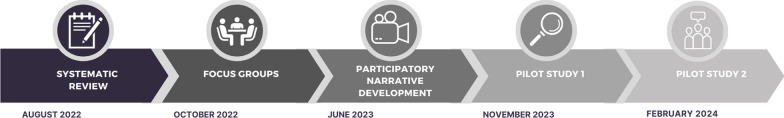
Overview of the five stages of the video development process

Ethical approval for completion of this study was obtained from the ethics committee of the Medical Faculty at the University Hospital Tübingen (reference 417/2022BO2, 418/2022BO2). An overview of the five stages of the video development process can be seen in Fig. 1, while a detailed explanation is provided below.

### Stage 1: systematic review

A systematic review was completed to determine the most common barriers and facilitators affecting ED treatment uptake and engagement, including a synthesis of the perspectives of patients with ED, their caregivers, and healthcare professionals. Further information and results of the systematic review have been published elsewhere (see [1]).

### Stage 2: focus groups with affected persons

#### Participants

Recruitment e-mails were sent to approximately 60 participants of previous ED research studies completed by the University Hospital Tübingen. A total of n = 8 participants attended one of two 90-min focus-group appointments at the University Hospital Tübingen.

#### Procedure

Following a short round of introductions, participants were provided with a list of barriers and facilitating factors regarding treatment uptake for EDs, as identified by the systematic review [1]. Participants were asked to identify which of these barriers and facilitators were relevant to their own treatment journey. A semi-structured group discussion followed, during which participants provided further insights into the most important barriers and facilitators of their treatment journey. Participants were further asked if there were any other important barriers and/or facilitating factors which were not included in the list. Written informed consent was obtained from all participants prior to begin of the focus groups. Participants received a compensation for their participation.

#### Analysis

A list of the barriers and facilitators that were reported as being important by participants was compiled and documented by the research team to determine how frequently each barrier and facilitator was identified. The group discussions were recorded and transcribed manually by a member of the study team. Selected quotes were chosen from the transcripts; however, the content of the transcripts has not been further analysed.

### Stage 3: participatory narrative development

#### Participants

A former and currently recovered patient with an ED was recruited as a lived experience representative to ensure that the patient narratives would remain relevant and relatable for affected persons.

#### Procedure

The current study utilised a collaborative approach to develop a series of evidence-based patient narratives. The methodological approach used here was created by the study team and has been detailed to provide one example of how patient narrative videos may be developed. In order to gain further insight into her personal experiences, the lived experience representative was initially asked to summarise her ED journey in a letter to her “former self”, including what motivated her to seek treatment and what information she wished she would have known prior to treatment. During a series of collaborative discussions, four key categories were developed on the basis of the lived experience representative’s personal experiences and the results of the systematic review [1] and focus groups (see Results). Using these four categories, two scripts were developed in cooperation with the lived experience representative with identical content, but with a variation of the intensity of the emotionality of the content, as defined by the lived experience representative. Throughout this process, it was decided that the lived experience representative would not disclose which ED symptoms or diagnosis she previously experienced in the scripts, but that she would instead discuss those aspects of the treatment journey which are shared by most patients with ED. Two further scripts were developed in cooperation with a psychotherapist with professional ED experience. The scripts for the psychotherapist contained the same content as those of the lived experience representative but were adapted to reflect a more professional point of view. In order to increase comparability, the psychotherapist was the same age and gender as the lived experience representative. Finally, two additional scripts were developed in which the lived experience representative discussed the same content presented in her initial two ED-focused videos, but with regard to a somatic condition unrelated to ED (i.e., a torn knee ligament). These scripts contained the same content as the lived experience representative’s scripts regarding her ED, however, the content was adapted to reflect the concerns, treatments, and need for social support of patients receiving treatment for this somatic condition.

A professional videographer was hired to film all six videos in June, 2023. Further editing was completed to ensure that all videos were comparable regarding duration, with the final videos being approximately six minutes in length. The lived experience representative received travel and accommodation reimbursement for the duration of the filming process.

### Stage 4: pilot study with participants without (current) eating disorders

#### Participants

Participants were recruited via the online mailing systems of the University Hospital Tübingen and the Eberhard Karls University Tübingen. Female students and employees of the University and University Hospital were invited to participate, provided they fulfilled the following inclusion criteria: minimum age of 18 years, sufficient German language skills, no current acute psychiatric disorder (e.g., suicidal ideation or psychosis).

#### Materials used

Participants completed a short demographic questionnaire including items regarding age, gender, nationality, and if they had any previous experiences with ED. A new questionnaire was developed by the research team for this study consisting of 11 items, in which participants were asked to rate the authenticity (e.g., Item 3: “I believe that the person in this video was personally affected by an eating disorder”) and empathy (e.g., Item 5: “I feel compassion for the person shown in this video”) of the person featured in the lived experience representative videos re. ED, the psychotherapist videos and the lived experience representative videos re. a somatic condition. Participants also rated the usefulness of the lived experience expert and psychotherapist videos (e.g., Item 9: “I think this video is helpful for people with eating disorders”) using a 5-Point Likert scale ranging from 1 = strongly disagree to 5 = strongly agree. Participants were further asked to provide an overall rating for each of the six videos using a 6-Point Likert Scale ranging from 1 = insufficient to 6 = very good. Participants completed the questionnaire immediately after watching each video. After watching both the low emotionality and high emotionality versions of each narrative video, participants were asked to indicate which version (low vs. high emotionality) they preferred. In addition, a subgroup of n = 5 participants was randomly selected by the study team and invited to participate in semi-structured interviews in order to gain additional insights into their perception of the videos, which may otherwise have been overlooked in the questionnaires, with particular focus on the authenticity, empathy, likeability, and usefulness of the videos as a whole, as well as which video/s they preferred the most.

#### Procedure

All data collection was completed in the Department for Psychosomatic Medicine and Psychotherapy at the University Hospital Tübingen. Participants were required to attend one study appointment with a duration of approximately 60 min, for which they received a compensation. All participants were asked to view and rate all six patient narrative videos created for this study. Participants were not supplied with any information regarding the research aims or how the videos differed from one another (i.e., different perspectives or emotionality). The order in which the videos were presented was randomised for each participant, both in regards to which perspective was seen first (i.e. lived experience re. ED, psychotherapist, lived experience re. a somatic condition) and which level of emotionality was seen first (i.e. low emotionality, high emotionality).

#### Statistical analyses

Data analyses were conducted using statistical software IBM SPSS Statistics Version 28 [21]. Authenticity, empathy, and usefulness scores were determined by calculating the mean of the respective items included for each domain in the questionnaire. A series of four repeated measures analyses of variance (ANOVA) were completed, wherein the Video Perspective (3 levels) and Emotionality of the Video (2 levels) were included as within-subject factors, and the authenticity, empathy, usefulness, and overall ratings for each of the six videos were assessed as dependent variables. Post-hoc tests were completed to further investigate pairwise comparisons between the three levels of the within-subject factor Video Perspective. The semi-structured interviews were recorded and transcribed manually by a member of the study team. Selected quotes were chosen from the transcripts to highlight individual feedback points provided by participants; however, the content of these transcripts has not been further analysed.

## Results

### Results from focus groups

**Fig. 2:**
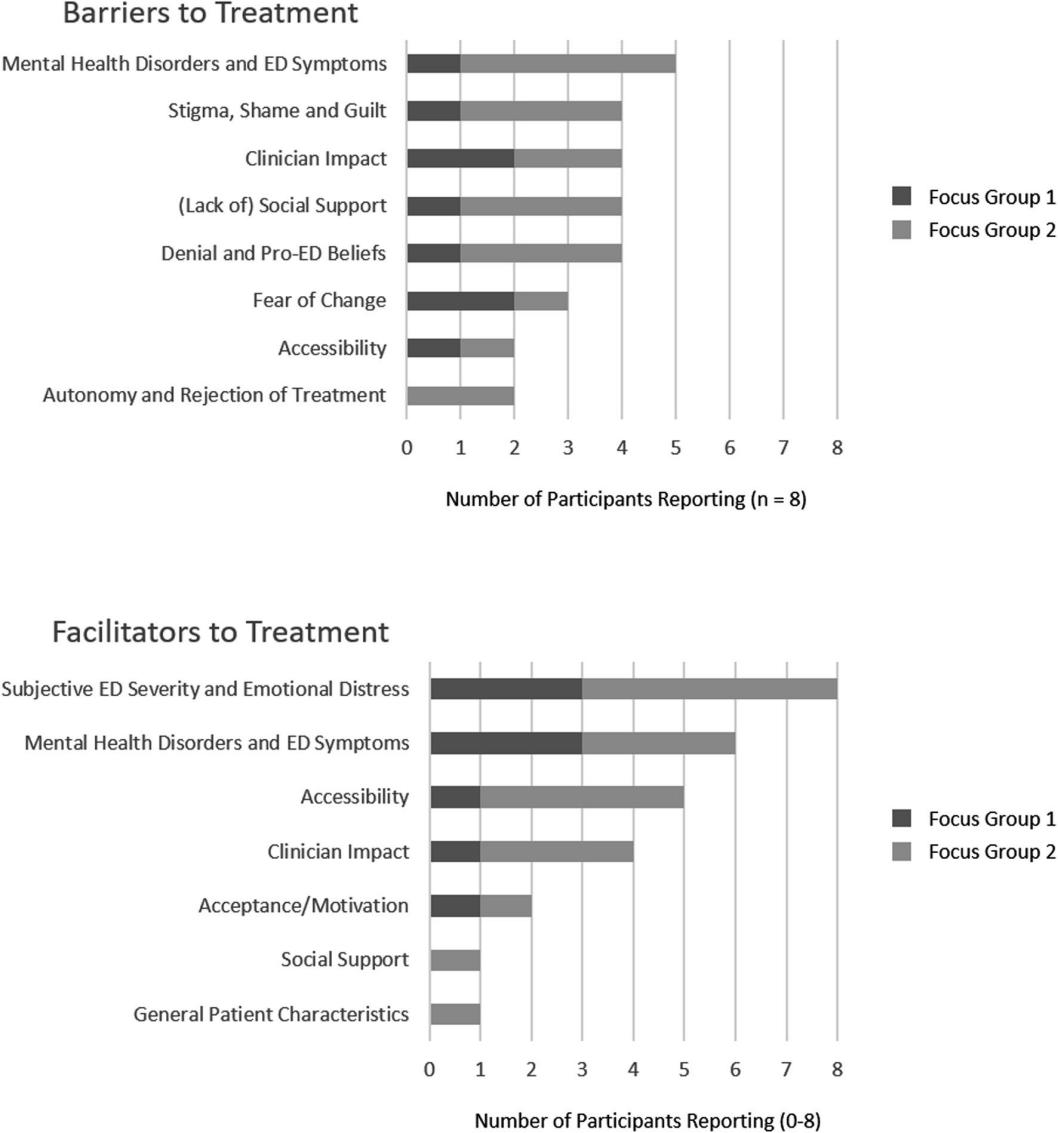
Barriers and Facilitators to Treatment, as reported by the Focus Groups (n = 8). Notes: ED = eating disorder

Two focus groups were completed between September and October 2022 with persons with ED residing in the area of Tübingen, Germany, so as to validate the results of the previously completed systematic review. The first focus group had a total of n = 5 participants, including both male and female patients with Binge Eating Disorder. The second focus group consisted of n = 3 female participants with Anorexia Nervosa or Bulimia Nervosa. A full overview of the results can be seen in Fig. 2. Participants of the focus groups reported that comorbid mental health disorders and ED symptoms (n = 5, 62.5%), feelings of stigma, shame, and guilt (n = 4, 50%), as well as the clinician impact (n = 4, 50%) were important barriers to seeking and/or engaging with treatment services. Participants further reported that a lack of social support (n = 4, 50%), as well as denial and pro-ED beliefs (n = 4, 50%) had a negative effect on their treatment. Meanwhile, all participants of the focus groups reported that the subjective ED severity and emotional distress were primarily responsible for their seeking and/or engaging with treatment services (n = 8, 100%). Other facilitators to treatment included comorbid mental health disorders and ED symptoms (n = 6, 75%), accessibility of treatment (n = 5, 62.5%), and clinician impact (n = 4, 50%).

Discussions in the focus groups allowed participants to elaborate on the individual barriers and/or facilitators which they experienced in their personal treatment journey. These discussions focused heavily on issues regarding accessibility and the local healthcare system, as well as experiences with stigma, shame, and guilt in relation to EDs. A selection of quotes from participants can be found in Table 1 to provide further insights.

**Table 1:** Selection of quotes from the focus groups discussing their experiences with “Stigma, Shame, and Guilt” in relation to their eating disorder

*"I somehow never managed to inform the people around me or get into those conversations, because for me I always saw it as a taboo subject and as a stigma"* - Participant 1, currently affected by Anorexia Nervosa *“In any case, with people who are overweight, it's always a case of, yes, it's your own fault, eat less, be more disciplined, erm, so in principle the blame is always put on the patient, so in my experience it's always the patient's fault”* - Participant 2, currently affected by Binge Eating Disorder *“It was a bit like, I wasn't sick enough, first of all through my own conviction, but also somehow from everyone else […] I don't know but there is such a hierarchy of eating disorders […] and as a man, now not only as a man but as a man in my personal experience I would hear 'well, he just likes to eat' or 'he just eats a bit too much' or things like that […]” - Participant 3, currently affected by Binge Eating Disorder*	

### Results from the participatory narrative development

**Fig. 3:**
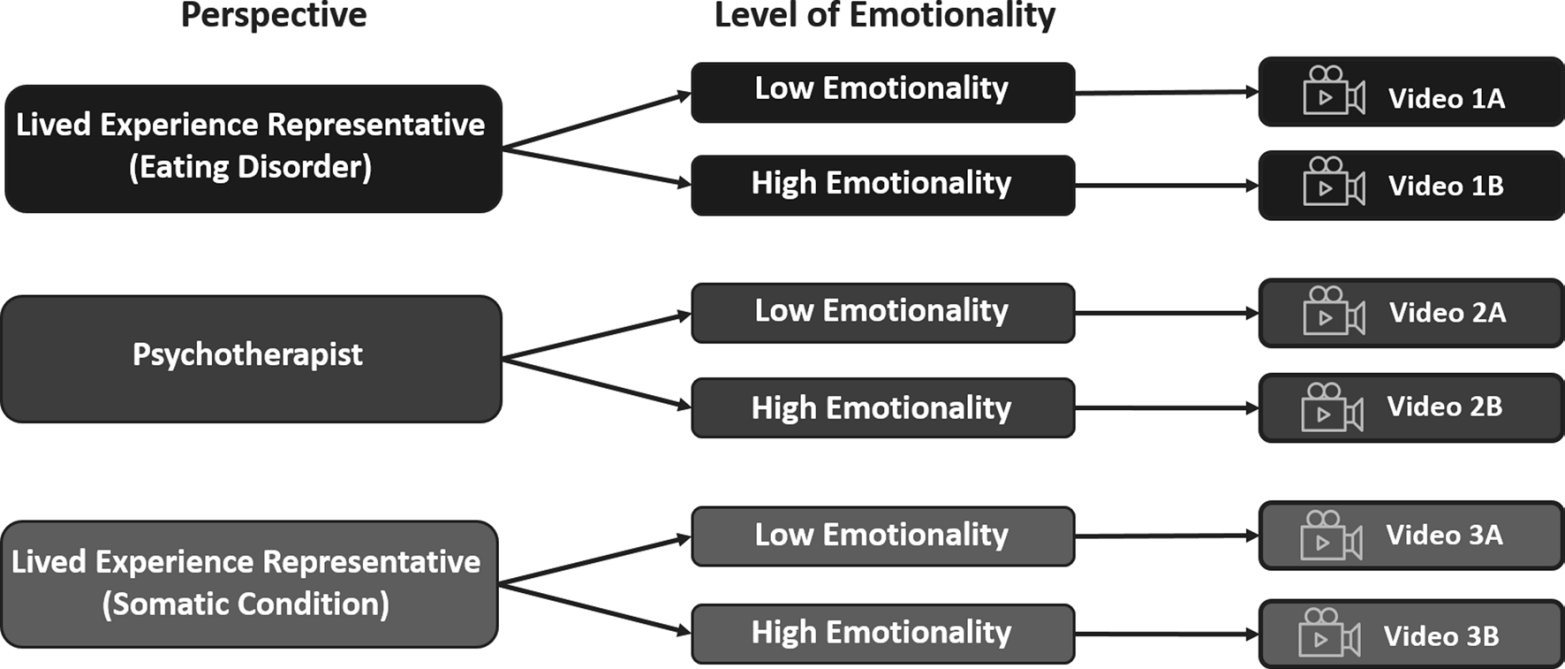
Overview of the six narrative videos

Six patient narrative videos were developed from the perspectives of a lived experience representative (Videos 1A and 1B), a psychotherapist (Videos 2A and 2B), and the lived experience representative reporting on a somatic condition unrelated to ED (Videos 3A and 3B; for an overview, see Fig. 3).

Each video consists of four key categories, based on a combination of the lived experience representative’s personal experiences and the results of the systematic review [1] and focus groups with currently affected persons:

1. The role of stigma, shame, and guilt as a barrier to treatment
2. The role of subjective distress caused by the ED as a facilitator for treatment
3. The importance of social support before and during treatment
4. Overall advice and encouragement for currently affected persons.

The level of emotionality was varied across the videos, so that a lower and a higher emotionality version was created for each perspective. To better demonstrate this, Table 2 shows the same excerpt of the script and how it is presented according to the different perspectives and levels of emotionality.

**Table 2:** Script excerpts according to perspective and level of emotionality

	Lower emotionality	Higher emotionality
Perspective: lived experience representative re. eating disorder	*“I had been suffering from an eating disorder for a few months and didn’t know how long I could continue living my life in this way, because I actually still had so many plans.”*	*“It was only when the eating disorder had a big enough impact on my life that I realized that I might be “sick enough” for treatment. Over the course of a few months my symptoms worsened, and I suffered a lot due to the eating disorder and the associated fears, rules, and loneliness.”*
Perspective: psychotherapist	*“Patients often spend several months struggling with the eating disorder, during which time they suffer increasingly and become unsure if they can continue living in this way.”*	*“Patients report that they will only admit to suffering from an eating disorder and being “sick enough” to receive treatment when the eating disorder has had a sufficient impact on their life. This usually takes some time, during which the patient’s physical health deteriorates and they suffer from the eating disorder and its associated fears, rules, and loneliness.”*
Perspective: lived experience representative re. a somatic condition	*“I spent a few months struggling with the pain and restricted mobility, during which time the psychological suffering I experienced kept growing. I didn’t know how I could continue living my life in this way.”*	*“It was only when the suffering had a big enough impact on my life that I thought I might be “sick enough” and should perhaps have surgery. Over the course of a few months my symptoms worsened, and I suffered a lot due to the pain and mobility restrictions. I didn’t know how I could continue living my life in this way.”*

### Results from the pilot study with participants without (current) eating disorders

In total, n = 19 participants were recruited for the initial pilot study. Participants identified as female (n = 18) or non-binary (n = 1), and ranged in age from 19 to 55 years (*M* = 26.00, *SD* = 8.29). The majority of participants were German citizens (n = 17), while the remaining participants were British (n = 1) or Chinese (n = 1) citizens residing in Germany. When asked about previous experiences with ED, n = 1 participant reported having had personal historical (but no current) experiences with an ED, while n = 9 participants reported having experiences through friends and/or family members.

#### Perspectives of the videos

*Authenticity* A significant main effect was found for the perspectives of the videos in regards to the authenticity ratings (*F*(1, 18) = 6.909, *p* = 0.003, *η*^*2*^ = 0.277). Post-hoc analyses showed that the lived experience videos re. ED were rated as significantly more authentic than the lived experience videos re. a somatic condition (*p* = 0.002). No significant differences were found between the lived experience videos and the psychotherapist videos (*p* = 0.106) or the psychotherapist videos and the lived experience videos re. a somatic condition (*p* = 0.060).

*Empathy* A significant main effect was also found for the empathy ratings of the videos (*F*(1, 18) = 9.301, *p* < 0.001, *η*^*2*^ = 0.341). Post-hoc analyses showed that the lived experience videos re. ED were rated as significantly more empathic than the psychotherapist videos (*p* = 0.008) and the lived experience videos re. a somatic condition (*p* = 0.004). No significant difference was found between the psychotherapist videos and the lived experience videos re. a somatic condition (*p* = 0.767).

*Usefulness* The results further showed a significant main effect for the usefulness ratings of the videos (*F*(1, 18) = 25.095,* p* < 0.001, *η*^*2*^ = 0.582), wherein the lived experience videos re. ED were rated as significantly more useful than the psychotherapist videos.

**Fig. 4:**
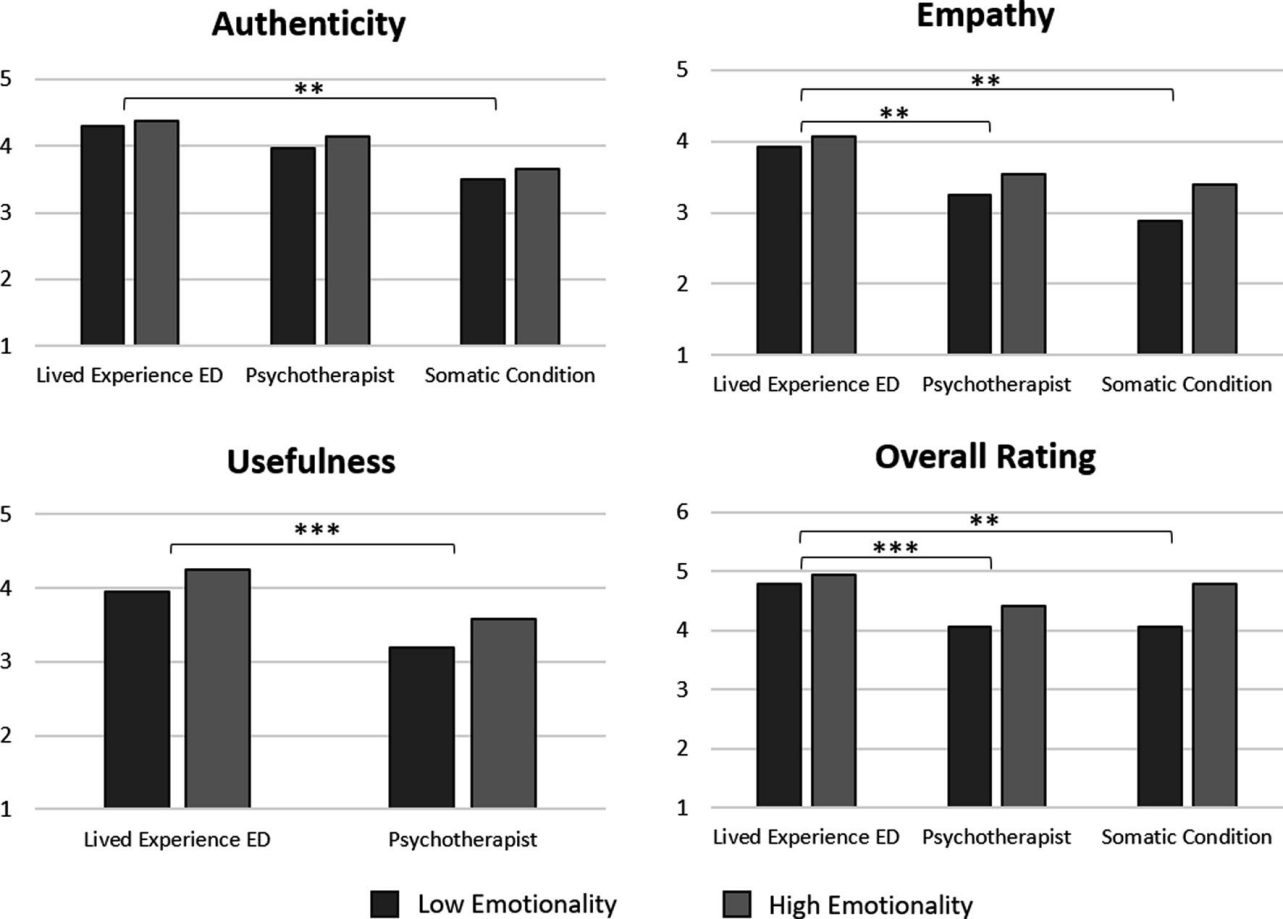
Comparison of the perspectives of the videos regarding authenticity, empathy, usefulness, and overall ratings. Note. For authenticity, empathy and usefulness y-axis scale: 1 = strongly disagree and 5 = strongly agree. For overall rating y-axis scale: 1 = insufficient and 6 = very good. *p = .05, **p = .01, ***p = .001

*Overall ratings* Lastly, the results showed a significant main effect for the perspective of the videos regarding the overall ratings of the videos (*F*(1, 18) = 6.018, *p* = 0.006, *η*^*2*^ = 0.251). Post-hoc analyses showed that the lived experience videos re. ED received a significantly better overall rating than the psychotherapist videos (*p* < 0.001) and the lived experience videos re. a somatic condition (*p* = 0.011). No significant difference was found between the psychotherapist videos and the lived experience videos re. a somatic condition (*p* = 0.446). A summary of these findings can be found in Fig. 4.

#### Emotionality of the videos

*Authenticity* No significant main effect was found for the emotionality of the videos regarding the authenticity ratings (*F*(1, 18) = 1.704, *p* = 0.208, *η*^*2*^ = 0.086).

*Empathy* A significant main effect was found for empathy ratings of the videos (*F*(1, 18) = 9.357, *p* = 0.007, *η*^*2*^ = 0.342), wherein the high emotionality videos were rated as more empathic than the low emotionality videos.

*Usefulness* A significant main effect was also found for the usefulness ratings of the videos (*F*(1, 18) = 4.490, *p* = 0.048, *η*^*2*^ = 0.200), wherein the high emotionality videos were rated as more useful than the low emotionality videos.

**Fig. 5:**
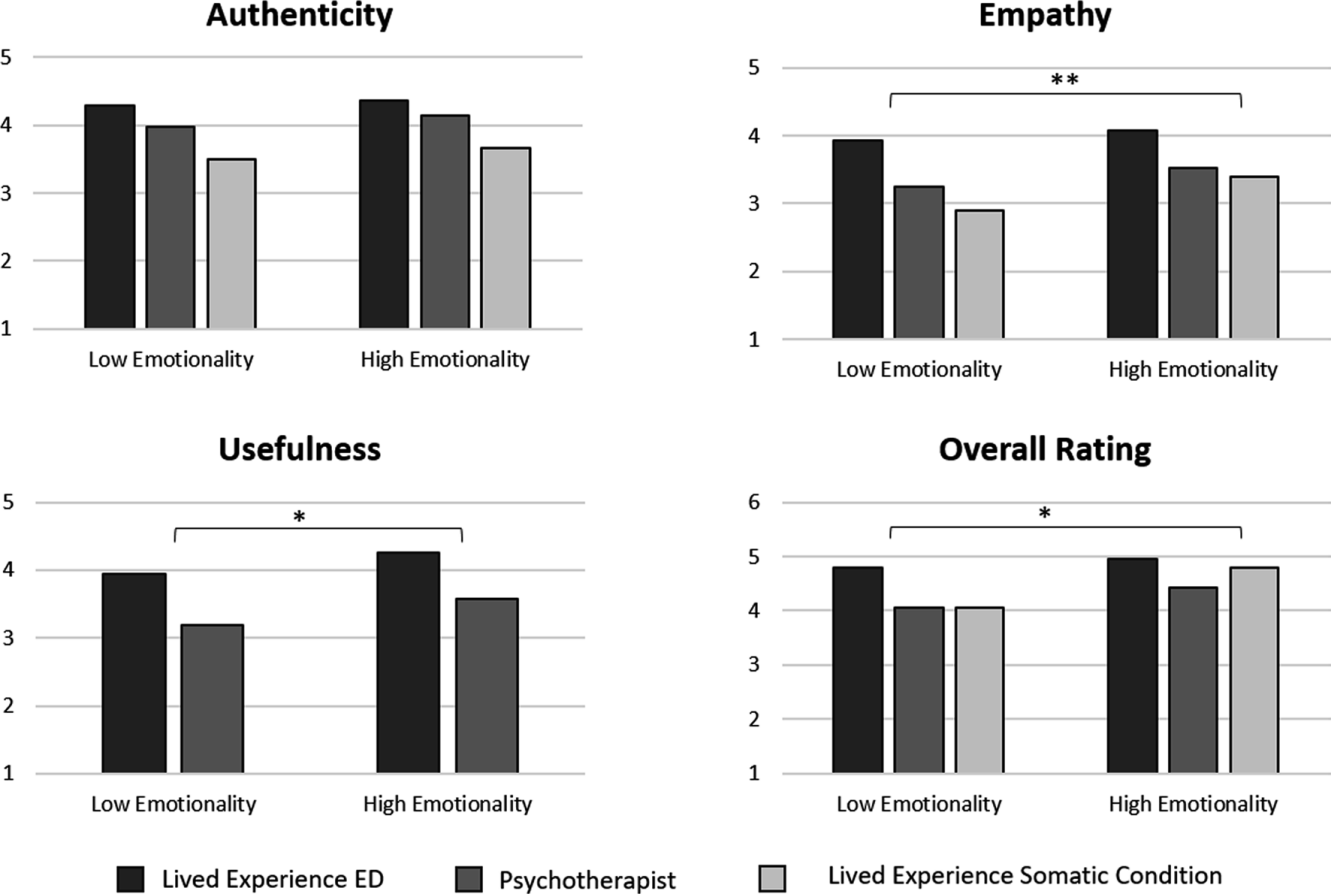
Comparison of the emotionality of the videos regarding authenticity, empathy, usefulness, and overall ratings. Note. For authenticity, empathy and usefulness y-axis scale: 1 = strongly disagree and 5 = strongly agree. For overall rating y-axis scale: 1 = insufficient and 6 = very good. *p = .05, **p = .01, ***p = .001

*Overall ratings* Lastly, the results also showed a significant main effect for the overall ratings of the videos (*F*(1, 18) = 5.586, *p* = 0.030, *η*^*2*^ = 0.237), wherein the high emotionality videos received a better overall rating than the low emotionality videos. When asked to explicitly state which level of emotionality they preferred, 68.4% of participants reported they preferred the videos with higher emotional content (i.e., Video 1B, Video 2B, and Video 3B). A summary of these findings can be found in Fig. 5.

#### Interaction effects

No significant interactions were found between the perspective and emotionality of the videos in regards to the authenticity (*F*(1,18) = 0.191, *p* = 0.827, *η*^*2*^ = 0.010), empathy (*F*(1,18) = 1.569, *p* = 0.222, *η*^*2*^ = 0.080), usefulness (*F*(1,18) = 0.175, *p* = 0.681, *η*^*2*^ = 0.010), or overall ratings of the videos (*F*(1, 18) = 2.661, *p* = 0.084, *η*^*2*^ = 0.129).

#### Follow-up interviews

Participants reported that the videos were generally authentic and empathic, and that they considered the videos as useful for patients with ED. Participants stated experiencing a variety of emotions while viewing the videos, including compassion, sympathy, and joy that the lived experience representative can present a positive outcome. When asked which videos they most preferred, n = 2 (40%) participants stated the lived experience videos re. ED, n = 1 (20%) stated the psychotherapist videos, and n = 2 (40%) stated they found both perspectives equally good. All participants reported that they found the lived experience videos to be more authentic than the psychotherapist videos. A selection of quotes from participants is provided in Table 3 to provide further insights.

**Table 3:** Selection of quotes from participants of the pilot study

"*[The videos] certainly made it clear what a great deal of suffering affected persons experience […] they especially made the thought processes that affected persons have a little more relatable for me*" - Participant 1, experiences with ED through friends/family “*A lot of different emotions were mentioned [in the videos], um, and I think that, um, maybe not every emotion will apply to everyone, but since there were quite a few, I can imagine that there will be something for everyone, um, that they will be able to identify with*” - Participant 2, no experiences with ED “*As someone who once suffered from an eating disorder, I definitely felt heard and included […] especially when it's another patient talking to you, it's like, okay, she's talking to me right now, there are two of us in this boat so we're sitting in the same place, we're at eye level*" - Participant 3, personal experiences with ED “*I can imagine that not only patients with eating disorders will be able to identify themselves [with the videos], but also patients with psychosomatic disorders or disorders or illnesses that are not visible*” - Participant 4, no experiences with ED	

## Discussion

The use of patient narratives for mental disorders, and especially ED, is a new methodological approach in clinical care and research. As such, it is difficult to find relevant information regarding the efficient development and implementation of patient narratives. The approach described in this paper is an example of how PPI can be effectively applied throughout the process of creating evidence-based patient narrative videos. This study combined five stages for the development of patient narrative videos: a systematic literature review, focus groups, participatory narrative development, and pilot studies evaluating the narrative videos.

Participants of the focus groups reported that in addition to the hindering effects of comorbid mental health disorders and individual ED symptoms, they experienced a considerable amount of stigma, shame, and guilt, which prevented them from seeking and/or engaging with treatment services. This confirms the findings of previously completed systematic reviews (e.g., [1, 2, 22, 23]), which also found that patients affected by ED most frequently reported stigma and shame as their biggest barriers to treatment. Participants of the focus groups further discussed that a lack of positive social support was a significant barrier to treatment, with many participants stating that they felt the need to “hide” their ED from others. The previously completed systematic review found similar results, whereby patients, caregivers, and healthcare professionals reported that the establishment of positive social support networks was the most important facilitator for treatment uptake and/or engagement [1]. A recent systematic review by Robinson et al. [24] similarly demonstrated a clear positive correlation between social supports and motivation to change among patients with a variety of ED, while a systematic review by Ramjan et al. [25] found a variety of positive outcomes including motivation to change among young people aged under 25 years among interventions which included social support as an adjunct to treatment. Other systematic reviews have also reported that a lack of positive social support may be a significant barrier to treatment seeking and/or engagement among patients affected by ED [2, 26]. Meanwhile, the severity of individual ED symptoms, and the resulting feelings of distress were discussed as primary motivation for treatment seeking among participants of the focus groups. Here the results varied somewhat from those of previous literature, in which the importance of individual ED symptoms as a barrier to treatment was represented, albeit significantly less pronounced [1]. This finding emphasises the importance of validating research results in the desired target population, to ensure that these remain relevant.

A total of six patient narrative videos were created on the basis of the results of the systematic review and the focus groups, using a collaborative approach with a lived experience expert. All six videos received positive ratings from a group of participants without (current) ED. Videos featuring the lived experience expert discussing her ED were rated as significantly more authentic than the lived experience videos re. a somatic condition, significantly more empathic than the psychotherapist videos and lived experience videos re. a somatic condition, and as significantly more useful than the psychotherapist videos and lived experience videos re. a somatic condition. The lived experience videos re. ED further received a significantly better overall rating than the psychotherapist videos and lived experience videos re. a somatic condition. The results of the interviews greatly mirror those of the post-intervention questionnaires. Although the majority of participants in this study had no personal experiences with ED, these findings nevertheless reflect those of prior studies in which content presented by former patients or “peers” was reported as significantly more empowering than the same content presented from the perspectives of healthcare professionals [15, 27]. Additionally, videos with higher emotionality were rated as more empathic and useful and received a better overall rating in comparison with videos with lower emotionality, indicating that high emotionality may be more empowering as well.

### Strengths and limitations

One of the greatest strengths of this study was its focus on collaborative approaches and inclusion of PPI. The lived experience representative featured in the patient narrative videos not only actively constructed the content of the videos, but has also been included as a co-author of this paper, thereby following current recommendations [28] to integrate lived experience authors into the research process. It should also be outlined that implementing PPI in mental health research is complex and requires the consideration of many issues, including ethical aspects and power dynamics within participatory research teams. For instance, a recent position paper by individuals engaging in PPI outlines the importance of reciprocity for positive outcomes in participatory research, including an active involvement of PPI representatives in the research process, reimbursement for time and expertise, and to be valued for contributions to the project [29]. Reciprocity in various ways can also prevent “pseudo-participation” of individuals with lived experience, however, for this to be avoided, there needs to be a structural foundation of PPI in society and research, including the opportunity to acquire funding for PPI [20].

A further strength of this study is the versatility of the patient narrative videos that were developed. The videos discuss aspects which may be relevant to all ED, so that affected persons can identify with them regardless of their ED diagnosis. Similar to the written narratives created by Dawson et al. [10], any possibly triggering content was removed, so that the videos remained purely motivational. Nevertheless, the videos are primarily focused on the experiences of one person and may therefore not be applicable to everyone. Consequently, there is a need for more diversity in future videos with regard to both the content and the person featured in the video.

A healthy sample of participants with no (current) ED was chosen for this initial pre-pilot for several reasons. As the patient narrative videos created for this study were brand new, the research team felt it was necessary to assess their feasibility and potential for any triggering content with an unaffected population, before showing these videos to currently affected persons. While the majority of these participants could not provide any personal ED experiences, they nevertheless provided a fresh perspective regarding the authenticity, empathy, and usefulness of the videos, and their feedback was used to determine whether these videos were ready for use in a clinical population. While this work underlines the high potential of authentic patient narratives, their evaluation by persons affected by ED remains unknown. Therefore, a further pilot study is currently being completed, this time with patients with EDs, to assess the effects of the patient narrative videos developed in this study on the treatment motivation and uptake of treatment services for patients EDs [30]. In this pilot study, the very same questionnaires are being used that were previously implemented in the present study with participants without a (current) ED, therefore allowing for a comparison of experiences between the groups (e.g. perceived authenticity of the videos).

Lastly, it should be noted that the qualitative methods utilised in this study were merely explorative. This pilot study was not intended to be a qualitative study per se. Rather, the purpose of the included focus groups and semi-structured interviews were to include the important insights of PPI to generate new hypotheses and ideas, as well as to ensure that no important feedback regarding the newly created materials were overlooked by relying solely on the results of quantitative surveys. Further studies with a stronger focus on qualitative methodology may therefore be useful to better reflect the experiences of viewing patient narrative videos such as those created in this study.

### Conclusion & perspectives

A series of evidence-based participatory patient narrative videos detailing the experiences of patients with ED were created in this study by utilising a multistep development process. An unaffected pilot sample rated the created videos as generally positive, with a clear preference for videos with content with higher emotionality. Based on these pilot results, the decision was made to utilize only those videos with higher emotionality (i.e., video Versions B) for a larger 4-arm pilot RCT that is currently being completed with persons currently affected by eating disorders [30].

The use of patient narratives for mental disorders, and especially ED, is a relatively new methodological approach in clinical care and research. As such, it is difficult to find relevant information regarding their efficient development and implementation. The approach used in this pilot study was one developed by the research team, and an example of how an evidence-based patient narrative may be constructed. We believe that it is important to document and share this process, so that other research groups may be able to use these findings for their own implementation and in order to stipulate more research in the field of patient narratives for clinical research on mental disorders.

## Data Availability

No datasets were generated or analysed during the current study.
